# Emerging Threats to Animals in the United Kingdom by Arthropod-Borne Diseases

**DOI:** 10.3389/fvets.2020.00020

**Published:** 2020-02-04

**Authors:** Arran J. Folly, Daniel Dorey-Robinson, Luis M. Hernández-Triana, L. Paul Phipps, Nicholas Johnson

**Affiliations:** ^1^Virology Department, Animal and Plant Health Agency (Weybridge), Addlestone, United Kingdom; ^2^Faculty of Health and Medicine, University of Surrey, Guildford, United Kingdom

**Keywords:** emerging infectious diseases, arboviruses, mosquito, tick, midge, vector, livestock, transmission

## Abstract

Worldwide, arthropod-borne disease transmission represents one of the greatest threats to public and animal health. For the British Isles, an island group on the north-western coast of continental Europe consisting of the United Kingdom (UK) and the Republic of Ireland, physical separation offers a barrier to the introduction of many of the pathogens that affect animals on the rest of the continent. Added to this are strict biosecurity rules at ports of entry and the depauperate vector biodiversity found on the islands. Nevertheless, there are some indigenous arthropod-borne pathogens that cause sporadic outbreaks, such as the tick-borne louping ill virus, found almost exclusively in the British Isles, and a range of piroplasmid infections that are poorly characterized. These provide an ongoing source of infection whose emergence can be unpredictable. In addition, the risk remains for future introductions of both exotic vectors and the pathogens they harbor, and can transmit. Current factors that are driving the increases of both disease transmission and the risk of emergence include marked changes to the climate in the British Isles that have increased summer and winter temperatures, and extended the period over which arthropods are active. There have also been dramatic increases in the distribution of mosquito-borne diseases, such as West Nile and Usutu viruses in mainland Europe that are making the introduction of these pathogens through bird migration increasingly feasible. In addition, the establishment of midge-borne bluetongue virus in the near continent has increased the risk of wind-borne introduction of infected midges and the inadvertent importation of infected cattle. Arguably the greatest risk is associated with the continual increase in the movement of people, pets and trade into the UK. This, in particular, is driving the introduction of invasive arthropod species that either bring disease-causing pathogens, or are known competent vectors, that increase the risk of disease transmission if introduced. The following review documents the current pathogen threats to animals transmitted by mosquitoes, ticks and midges. This includes both indigenous and exotic pathogens to the UK. In the case of exotic pathogens, the pathway and risk of introduction are also discussed.

## Introduction

The threats posed to public health from vector-borne diseases are a subject of considerable investigation, particularly as changes to the climate may increase such threats (1, 2). Less attention has been paid to the threat to animals, and by animals, we include livestock, domestic pets and wildlife. To address this, and with a focus on the United Kingdom (UK), we have compiled both an inventory that includes the actual and potential vector-borne diseases that are a threat to animals and assess the risk they pose. The impact of vector-borne diseases to animals is varied. Many of the diseases considered are zoonotic so infection may not cause overt disease in animals, but their infection provides a pathogen reservoir that could eventually affect the human population. Where disease results from infection, this can lead to morbidity and mortality. In the case of livestock, certain diseases are considered notifiable (defined below) and could result in cessation of trade with other countries. This will have an economic impact that could take years to resolve and is a powerful motivating force to control disease outbreaks and limit the resulting losses. For wildlife, the emergence of disease in naïve host species could lead to a decline in population numbers that combined with anthropogenic factors that reduce available habitat or reproductive activity, could threaten species with extinction.

The definition of a notifiable disease is any disease that is required by law to be reported to a competent authority, usually governmental. The primary purpose of this, whether from a human or governmental perspective, is to prevent disease spread. In the UK the competent authority for human diseases is Public Health England within the Department of Health. For animals, this is the Department for Environment, Food and Rural Affairs (Defra). Devolution has led to the development of agencies that investigate animal disease on behalf of the devolved governments for example the Scottish Agricultural College (SAC) in Scotland and the Agri-Food Biosciences Institute (AFBI) of Northern Ireland. Veterinary investigations of livestock, poultry and equines are carried out by the Field Services Division of the Animal and Plant Health Agency (APHA). This is supplemented by veterinary services offered by university-associated Veterinary Schools of which there are six in England and two in Scotland. Other organizations offer veterinary support including The Pirbright Institute (Livestock Virology), the Institute of Zoology, and the Animal Health Trust. Domestic pets are usually dealt with by private veterinary surgeons (PVS). Wildlife monitoring, surveillance and health can involve all the above organizations and a large number of charitable bodies.

Some of the diseases discussed below are endemic. However, many are not and understanding how they can enter the UK is a key step in understanding the risk of emergence. For vector borne diseases there is the added concern of the vector and its distribution. Like diseases, not all potential vectors are present in the UK. The routes of pathogen entry are often termed pathways of introduction. For vector borne diseases this could take the form of an infected human or animal. For notifiable diseases some screening of animals for disease prior to movement is usually required to prevent importation of infected livestock or domestic animals. Another pathway is the introduction of the vector of a particular disease. For midges, wind movements can lead to their introduction. For other vectors, such as mosquitoes and ticks, passive introduction, for example the importation of dogs infested with *Rhipicephalus sanguineus* s.l. ticks, does occur (3). Another pathway is through the movement of wildlife. For the UK, separated from the mainland of Europe by the English Channel, the main risks are associated with pathogens and vectors that are associated with migrating birds. Although not conclusively shown, it is possible that viraemic birds could expose the indigenous mosquito population to a number of viruses that would then threaten public and veterinary health. Alternatively, migrating birds are occasionally infested with ticks and this can be a route for exotic ticks, such as *Hyalomma* spp. to enter the UK. In addition, invasive mosquito species have established across Europe and are spreading further north. This spread into countries in Western Europe has been the source for importation of the Asian tiger mosquito (*Aedes albopictus*) into southern England, probably through passive transport in cars or lorries (4).

One overarching factor that could affect the risk of vector-borne disease is the impact of climate change. There is general consensus that average temperatures will rise in the UK over a timescale measured in decades. However, the impact this will have on arthropod populations is unclear as higher temperatures alone are not the only critical factor for many vector life-cycles. Both mosquitoes and ticks require moisture, mosquitoes for larval development and ticks to avoid desiccation during maturation phases between feeds. In addition, extremes of weather, such as storms or drought could have a negative effect on vector populations. One possible scenario is that indigenous vectors may become more abundant and active for longer in the year. The UK could also become colonisable to exotic species. This could lead to a larger diversity of tick, mosquito or midge species, and the potential introduction of new vectors, such as sandflies. Consequently, understanding the existing diversity and distribution of vectors, and how this evolves in response to climate change remains critical to predicting future disease threats.

Arthropod vectors are usually associated with nuisance biting. For mosquitoes there are currently no diseases that indigenous UK species transmit to humans. However, malaria was endemic in marshy areas in the east of England until the start of the twentieth century (5). Despite reintroductions after both World Wars, the parasite was eliminated, as it was from the rest of Europe until recently (6). For ticks there are a larger number of indigenous diseases of animals associated with bites, particularly from the most common tick species in the UK, *Ixodes ricinus*, which transmits louping ill virus, *Babesia divergens* and *Anaplasma phagocytophilum* in the UK. For humans, tick bites from this species can result in Lyme disease. Cases of Lyme disease have also been reported in dogs (7) and pet dogs have been proposed as a sentinel for disease risk (8). Finally, biting midges are a major vector for a number of high-impact veterinary diseases. The following sections describe and discuss the actual and potential threats to animals within the UK grouped by arthropod vector.

## Mosquitoes and Mosquito-Borne Diseases

**Figure 1:**
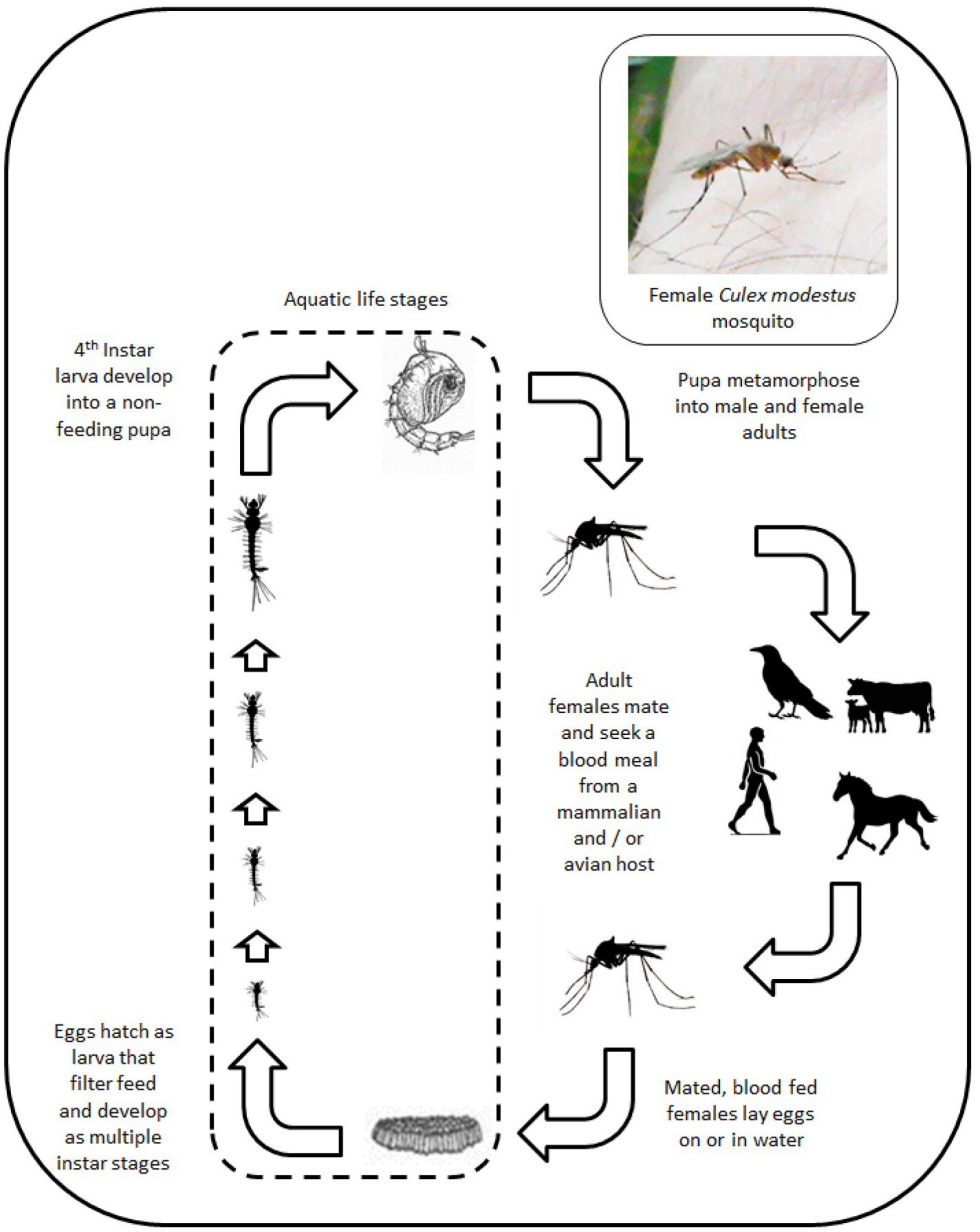
Schematic showing the life cycle of mosquitoes.

There are over 30 mosquito species present in the UK (listed in Table S1). All obtain nutrition through feeding on vertebrate hosts (Figure 1). Potentially the most important from a disease transmission perspective is the species *Culex pipiens*, a vector for a number of viruses including notifiable viruses, such as West Nile virus (WNV). *Cx. pipiens* is a species complex containing a number of morphologically similar forms with different bionomic properties that influence virus transmission (9). A key property of *Cx. pipiens* is its abundance across many areas of the country that put many areas at risk of virus spread. Other species, such as *Aedes detritus*, have also been associated with transmission of a number of viruses (10–12). However, in contrast to *Cx. pipiens*, its distribution is limited to coastal sites and estuaries because of its requirement for salt water for oviposition and larval development.

A key feature of mosquitoes within the UK is their seasonality. Activity begins in early spring but most species only become abundant during the summer months (13). Activity declines during autumn and there is a complete cessation of activity with species over-wintering in a variety of forms (desiccated eggs, diapaused larvae and mature females). This restricts the period over which mosquito-borne transmission can occur and may be one of the reasons why there has been no evidence of autochthonous mosquito-borne virus transmission in the UK since the inadvertent introduction of both yellow fever virus and its vector, *Aedes aegypti*, in 1865 (14).

Another key feature is the host feeding preference of mosquitoes for a vertebrate host. All species within the UK require a blood meal to provide sufficient nutrition to enable egg development and maturation. Mosquitoes generally target either a mammalian host or an avian host with this having clear implications for the ability to transmit viruses between non-conspecifics. Some mosquito species that feed on multiple hosts can act as a “bridge” vector enabling transmission of a virus that normally replicates in birds being transmitted to humans and livestock. A number of previous studies have confirmed that mosquitoes feed on a range of livestock and wildlife (15–19). In the case of some mosquito species including *Culiseta annulata* and *Anopheles messeae*, they appear to feed exclusively on large ruminants. Others, such as *Anopheles atroparvus*, are more opportunistic. The recent observation of *Culex modestus* in the Kent Estuary (20), a bridge vector for WNV in mainland Europe, has raised concerns that this could provide a vector population if the virus was introduced, although surveillance has not detected WNV in this mosquito population to date (21).

### Threats From Mosquito-Borne Viruses Present in Europe

The most prominent disease threat to UK public and animal health is from those viruses that are already present in Europe as these could be more readily introduced by migratory birds. Predominantly these are flaviviruses, which are known to be transmitted by arthropod vectors and can cause disease in wildlife, livestock and in some cases humans. Below we expand on some of these economically important flaviviruses (Table 1). A number of these are currently active in Europe and capable of causing disease in wildlife, livestock and humans (22).

**Table 1:** Bird associated viruses within the genus *Flavivirus*.

**Virus**	**Distribution**	**Susceptible vertebrates**
Bagaza virus (BAGV)	Spain, sub-Saharan Africa, India	Partridge, pheasants
Israel turkey meningo-encephalitis virus (ITV)	Israel, South Africa	Turkey
Japanese encephalitis virus (JEV)	Asia	Humans, pigs, equids
Louping ill virus	United Kingdom	Sheep, cattle, grouse
St. Louis encephalitis virus (SLEV)	North America	Humans
Tembusu virus (TMUV)	Asia	Duck, goose, chicken
Usutu virus (USUV)	Africa, Europe	Passeriformes, Strigiformes
West Nile virus (WNV)	Africa, Europe, Americas, Asia	Passeriformes, Accipitriformes, humans equids

### West Nile Fever (WNF)

**Figure 2:**
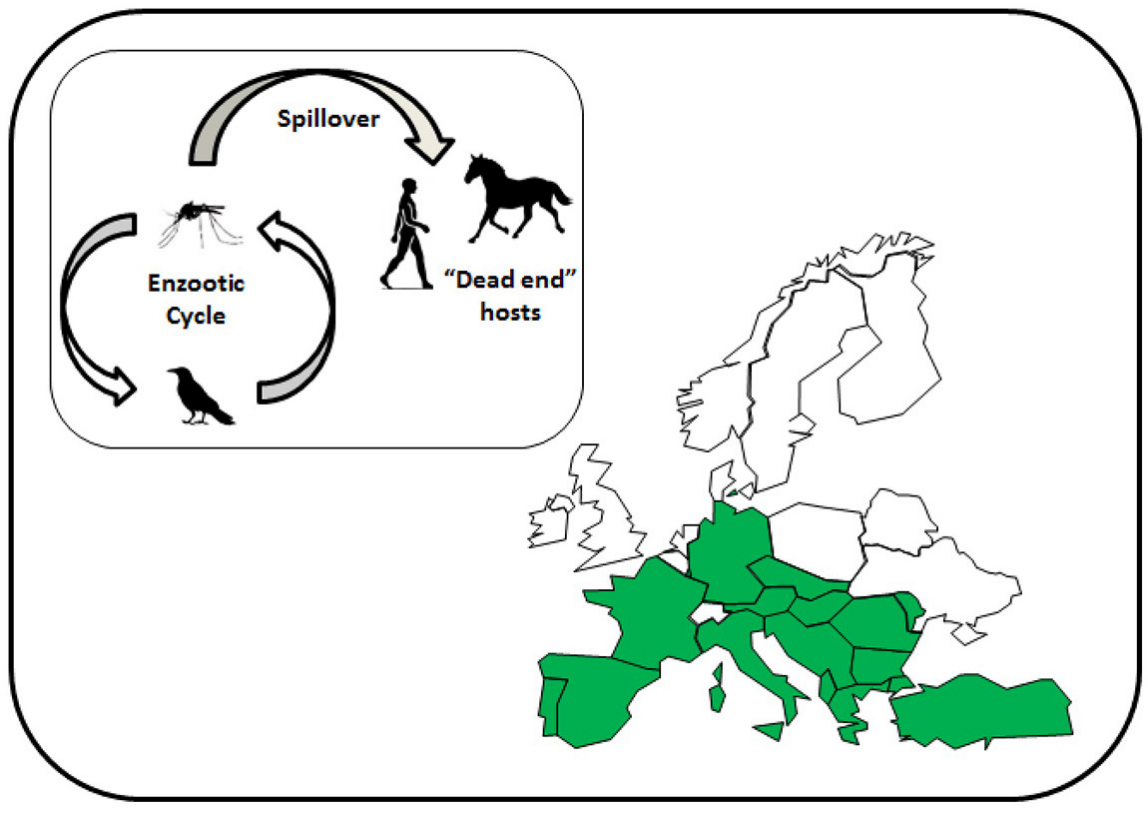
Map of Europe showing the countries affected by West Nile virus to 2018 (marked in green). Inset shows the reservoir cycle between mosquitoes, mainly *Culex* species, and birds, and spillover into mammalian species.

Until the start of the twenty-first century, WNV caused sporadic outbreaks in Europe that affected both human and equine populations, but rapidly resolved once mosquito activity declined at the end of the summer. Notable outbreaks occurred in the Camargue region, France, in 1962 (23) and Bucharest, Romania, in 1996 (24). Figure 2 shows the European countries that have reported cases of WNV in humans and/or horses. Domestic poultry have been affected in Europe (25), but this is not a common observation considering its prevalence and transmission by ornithophilic mosquitoes. A more common observation is disease within birds of prey (26) and these are a distinctive target for syndromic surveillance.

Over the past two decades, outbreaks due to various lineages of WNV have increased to the point where the virus is now considered endemic in some countries of southern Europe, resulting in regular outbreaks in particular regions, such as the Po Valley in Italy and the Camargue in France. This distribution changed in 2018, a year that experienced a particularly warm summer with above average temperatures for a number of months. Possibly as a result, WNV cases occurred in Germany at latitudes considerably further north than reported in previous years (27). Whether WNV establishes at these northerly latitudes and continues to spread will likely depend on the climatic conditions across northern Europe over subsequent summers. However, surveillance is critical to provide early detection of virus in arthropod and avian reservoirs prior to transmission to humans (28, 29).

WNV is notifiable in the UK in horses. The virus has not been detected in the UK although a seropositive horse (30) has been reported resulting from importation. However, the risk of introduction was recognized and a protocol put in place to investigate suspected cases without unnecessarily implementing the full range of veterinary control measures (31). Horses are considered a dead-end host, due to low viraemia and consequently are incapable of infecting other horses directly or infecting potential vectors. A risk assessment published by the Department for Environment, Food and Rural Affairs, identified eight potential pathways (Table 2) that could lead to the introduction of WNV into the UK (32). Overall, the greatest risk was associated with introduction by migratory birds and even this was considered “very low,” being defined as very rare but cannot be excluded. The caveat to that has been a dramatic change in the distribution of WNV in Europe that has led to infected birds being detected in northern Europe, reducing the potential distance that birds would need to migrate across to reach the UK.

**Table 2:** Risk pathways for the introduction of West Nile virus into the UK [adapted from Defra (32)].

**Pathway**	**Risk**
Introduction by migrating birds	Very low
Importation by legal trade in horses	Very low
Importation by legal trade in biological materials (equine semen, ova, embryos)	Negligible
Importation by legal trade in poultry	Negligible
Importation by legal trade in non-equine/non-avian species	Negligible
Illegal importation of infected animal	Impossible to quantify
Importation of infected vector	Very low
Air-borne movement of vector from continental Europe	Negligible

### Israel Turkey Encephalitis (ITE)

Israel Turkey encephalitis virus (ITV), the causative virus of ITE, was first reported in 1960 following descriptions of a neuro-paralytic disease of turkeys (*Melaeagris gallipavo*) in Israel (33). In addition, Bagaza virus (BAGV) was isolated from *Culex spp*. mosquitoes in the Central African Republic (34) and has since been detected across sub-Saharan Africa and India (35). Interestingly, genomic sequence analysis has shown that these viruses are very closely related flaviviruses, to the point where they are effectively the same virus species. The repeated isolation of the BAGV in mosquitoes from countries in Africa suggests that mosquitoes are the vector, a feature shared with many viruses within the genus. BAGV was detected in Europe in 2010 following the death of large numbers of red-legged partridges (*Alectois rufa*) in southwestern Spain (36) and was coincident with cases of WNV in horses in the region. Common pheasants (*Phasianus colchicus*) were also affected during the epizootic. The source of the introduction, presumably from Africa, was not identified and there have been no further reports of disease in Europe, although seroprevalence studies have suggested that the virus continues to circulate in wild bird populations in Spain (37) and thus continues to present a risk of disease to poultry if it spreads more widely.

### Usutu Virus Infection

Disease caused by Usutu virus (USUV) has not been defined into a single disease entity. Infection in birds can lead to a range of disease signs and at necropsy virus is found throughout the organs of the infected animal (38), while infection in humans is rarely associated with disease. The virus is a flavivirus closely related to WNV that exists in a reservoir cycle between *Culex* spp. mosquitoes and birds. Unlike WNV, USUV is not particularly pathogenic in mammals, although occasional human infections are reported, but infection does appear to be more virulent for avian species (39). The first reports of USUV date back to the late 1990s and retrospective analysis of bird samples has found evidence for its introduction into Italy in 1996 (40). Various strains of the virus rapidly established in parts of the Mediterranean Basin and have been repeatedly detected during surveillance for WNV (41). The viral strains have also spread north, being detected in Germany (42) and Belgium (43) often associated with increased mortality in species, such as the blackbird (*Turdus merula*). Vector competence studies have shown that *Culex pipiens* mosquitoes from the Netherlands are highly competent to transmit USUV (44) that does not appear to be reflected by those present in the UK (45). However, the introduction of USUV by short distance avian migrants from the European mainland is possible, especially during the summer months, and justifies limited surveillance in target bird species (46). The major threat of USUV would be to avian species abundance and diversity, as infection may reduce populations of susceptible species.

### Infections Caused by Other Pathogens in Europe

Two further mosquito borne viruses that are implicated in disease in animals have been reported in Europe. Batai virus (BATV) is an orthobunyavirus related to Schmallenberg virus (see below). Repeated isolation of BATV suggest that mosquitoes are a competent vector for the virus (47, 48) although an association with a particular species has not been confirmed. Also the evidence that the virus causes disease in livestock is equivocal although serology studies in Germany detected evidence of infection (49) and BATV was recently implicated in the death of a harbor seal (50).

Sindbis virus (SINV) is an alphavirus that is transmitted between birds and ornithophilic mosquitoes. It is one of the most widespread viruses with evidence for its presence in Europe, including Scandinavia, where it causes a mild febrile illness and arthralgia in humans called Ockelbo in Sweden. SINV has also been detected in Asia and South Africa. It is assumed that birds are refractory to disease, although surveillance occasionally detects the virus in bird tissues (51). There is also evidence that SINV can cause neurological disease in African horses (52), although this has not been observed in Europe.

### Canine Heartworm

Canine heartworm, also known as subcutaneous dirofilariosis, is caused by the parasitic worms *Difilaria repens* and *D. immitis* (53). Immature microfilariae circulate in the bloodstream where they can be taken up by mosquitoes and transmitted to a new host. The adult form migrates to muscular tissue where they remain, eventually leading to disease. In the early 2000s, the distribution of *D. immitis* in Europe was associated with countries around the Mediterranean Sea (54), but infections have been documented in the UK, likely following import from mainland Europe (55). Recent surveys of PVS in Western Europe provide anecdotal evidence that cases are on the increase (56), related in part to the increasing number of dogs being taken on holidays in southern Europe (57).

## Significant Mosquito-Borne Viruses Affecting Animals Globally

### Japanese Encephalitis

Japanese encephalitis virus (JEV) is a zoonotic virus that is found throughout Asia. Like WNV and USUV, the virus persists in a bird-mosquito cycle that can spill-over into human and livestock populations. In contrast to the other viruses, pigs can act as a vertebrate reservoir host for JEV. As its name suggests it causes severe encephalitis in humans often leaving the patient with long term neurological deficit. It is also an economic disease of pigs causing abortion, still-birth and death in piglets (58). A range of *Culex* species transmit the virus, particularly *Cx. tritaeniorhynchus*, a species found in south east Europe (59). A number of recent reports have presented evidence for JEV in Europe (60, 61). However, these results are based on detection of partial genomic sequences not a complete genome, and live virus has not been isolated and as such there is some controversy over whether these are genuine cases of infection. If they are confirmed, it would represent a dramatic translocation of the virus. However, a single case of JEV has been reported from Africa in a human co-infected with yellow fever virus (62). Overall, the threat posed by this virus to the UK is low, although continued increase in air travel from Asia could lead to viraemic humans arriving in Europe where indigenous mosquitoes are competent to transmit JEV (10, 63, 64).

### Rift Valley Fever

Rift Valley fever (RVF) is a zoonotic disease of ruminants causing sporadic outbreaks among livestock caused by the Rift Valley fever virus (RVFV). RVF occurs across much of sub-Saharan Africa (65). Transmission is facilitated by bites from infected mosquitoes, although humans can become exposed through contact with infected carcases. In sheep and cattle, disease is initially a short-term febrile illness progressing to jaundice, hepatic failure, and hemorrhagic disease. Mortality is severe in juvenile animals reaching 90% in some outbreaks and high rates of abortion and neonatal malformation are common. Significant outbreaks have affected countries of North Africa and the translocation of infected animals has led to RVF being introduced into the Arabian Peninsula in 2000 (66–68). Other examples of its transmission beyond the African mainland include its emergence in Islands of the Indian Ocean including Madagascar (69) and Mayotte (70). In addition, serological studies have suggested that RVFV may be circulating in Turkey (71) and Iran (72). To date there has been no evidence of RVFV introduction into Europe, although some researchers have speculated that this is likely based on previous examples of translocation out of Africa and a number of studies have shown that mosquito species in Europe are competent vectors for the virus (12, 73). With the possible exception of human travel from Africa, it seems unlikely that RVFV could be introduced inadvertently in livestock or livestock products to the UK due to paucity of such trade at the current time. However, an increase in livestock trade with Africa or its introduction into mainland Europe would change this assessment. However, there is currently concern that RVFV could be introduced into the United States (US) and Europe (74).

### Saint Louis Encephalitis

St. Louis encephalitis virus (SLEV) is a flavivirus that appears to occupy the same ecological niche in the New World that WNV occupies in the Old. Indeed, when WNV emerged in North America in 1999 the initial cases were suspected to be infected with SLEV. Similar to WNV the primary transmission cycle of SLEV is between mosquitoes and birds, although mammals may also contribute. In addition, the virus may cause sporadic outbreaks of human encephalitis throughout North and South America. Phylogeographic investigations have suggested that SLEV emerged in the seventeenth century in Central America and been translocated by bird migration (75). Serological surveys suggest that livestock can be infected asymptomatically with SLEV (76) and there has been a report of a horse with neurological disease associated with infection with the virus (77). Critically, there is currently no evidence for SLEV infection outside of the Americas.

### Equine Encephalitis

The New World also hosts a number of zoonotic alphaviruses that cause encephalitis in humans and horses. These are collectively termed the equine encephalitides and the complex is composed of three viruses: Eastern equine encephalitis virus (EEEV); Western equine encephalitis virus (WEEV); and Venezuelan equine encephalitis virus (VEEV). Each is widely distributed, transmitted by a range of mosquito species and all viruses cause severe disease in equids and humans (78). As with SLEV, there has been no evidence for these viruses outside of the Americas despite extensive intercontinental transport of horses and the risk of introduction is considered negligible.

### Duck Egg-Drop Disease

Tembusu virus (TMUV) was first isolated in mosquitoes in Malaysia in 1955, and subsequently shown to cause encephalitis and growth retardation in chicks (79). Birds are the natural amplifying host and a number of wild species have been identified as playing a role in TMUV persistence. Interest in the virus has increased in recent years as it has been demonstrated as the causative agent of duck egg-drop disease in China (80). The virus has only been detected in South-east Asia and is not considered a threat to Europe currently.

### The Threat Posed by Invasive Mosquito Species

Non-native or invasive mosquito species have had a dramatic impact on public health in Europe. The introduction and spread of the Asian tiger mosquito (*Aedes albopictus*) in particular has been a major factor in outbreaks of chikungunya virus in Italy, and repeated outbreaks of dengue fever in southern France (81). Surveillance for invasive mosquitoes in the UK is conducted by Public Health England (82) and there have been a number of detections in England in recent years (83). The impact on animals from the introduction of invasive mosquito species is uncertain and there is little evidence from Europe that the establishment of such mosquitoes has led to increased disease prevalence in animals. The feeding preference of *Aedes albopictus* is varied depending on the availability of potential hosts (84, 85) and there are reports of the species feeding on cattle (86, 87). However, there is no evidence that were *Ae. albopictus* to establish in the UK, there would be greater risk of disease transmission to livestock or domestic animals.

## Ticks and Tick-Borne Diseases

**Figure 3:**
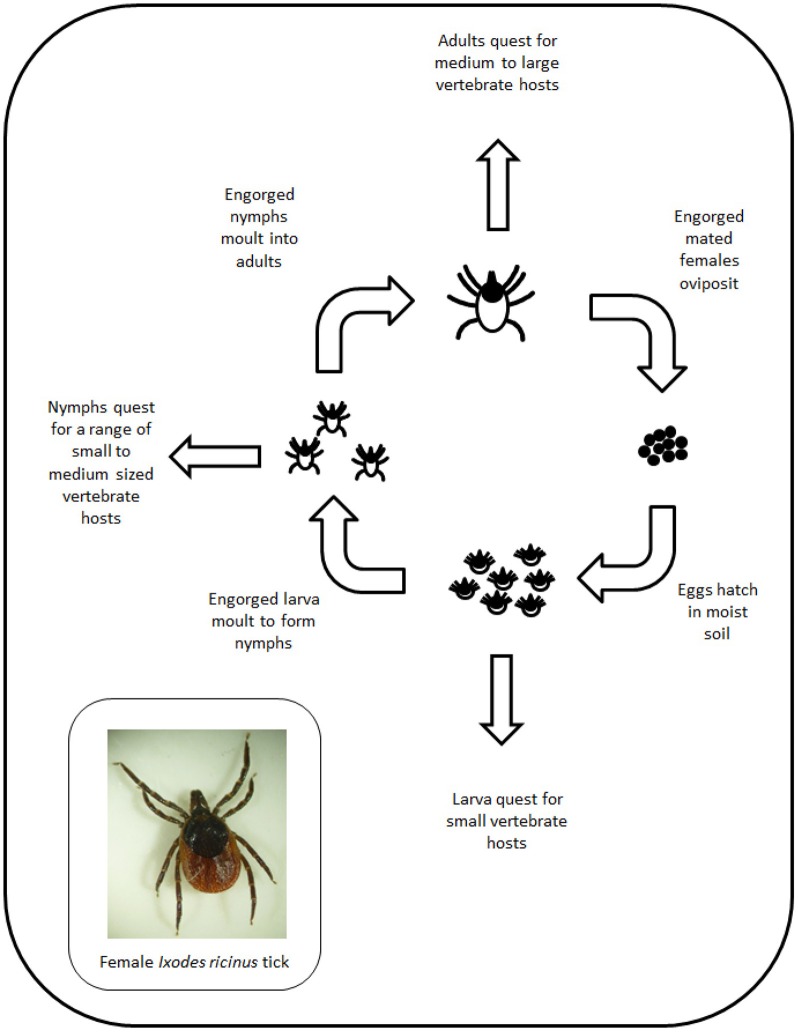
Schematic showing the life cycle of *Ixodid* ticks.

There are over 20 species of ticks indigenous to the UK (see Table S2) and all acquire nutrition through feeding on vertebrate hosts (Figure 3). Surveillance for ticks in the UK indicates that the species most often associated with tick bites to humans is the common sheep tick *Ixodes ricinus* (88, 89). Other species that feed on livestock but show limited geographical distribution include the ornate cattle tick (*Dermacentor reticulatus*) (90) and the red sheep tick (*Haemaphysalis punctata*) (91).

### Louping Ill

Louping ill virus (LIV) is the only indigenous tick-transmitted virus present in the UK. The disease results from viral encephalomyelitis, mainly affecting sheep, which show signs of neurological impairment including incoordination, altered gait and ataxia. Other mammals can be infected although such cases are rare. Of further economic significance is the susceptibility of red grouse (*Lagopus lagopus scoticus*) to infection (92). LIV occurs in upland areas of the British Isles (93, 94) with sporadic reports of disease in sheep from the west of Scotland, Cumbria, Wales, and Devon.

Louping ill is classified within the family *Flaviviridae* and genus *Flavivirus*, and is closely related to tick-borne encephalitis virus, a virus found across Eurasia causing disease in humans rather than livestock. The disease louping ill has been observed in sheep for centuries, but it was not until the late 1920s that the infectious agent was isolated from the central nervous system of sheep showing disease signs and demonstrated, through filtration, to be a virus (95). Shortly after this, the role of *Ix. ricinus* ticks in disease transmission was established (96). Since that time, most research has been directed at understanding the susceptibility of particular mammal species to LIV infection (97, 98) and the interaction of the tick vector, wild mammals and livestock in maintaining the virus within the upland ecosystem (99). Ticks can also be infected with LIV through co-feeding (defined as feeding in close proximity to another infected tick), without infection or viraemia in the host (100). This is thought to contribute to the persistence of LIV even when control measures in sheep, such as vaccination, are applied. Experimental studies in support of field observations have shown that duel infection with LIV and *Anaplasma phagocytophilum* (see below) can increase the severity of disease in sheep (101).

In addition to vaccination, alternative control measures include acaricide treatment of livestock and habitat management as means of preventing tick feeding and suppressing tick numbers, respectively. The identification of certain wildlife species that promote LIV persistence in upland areas (102) has led to the controversial management practice of culling mountain hares as a means of controlling tick abundance.

A number of viruses related to LIV are present in Europe. These are rarely reported but have very similar properties to LIV, including transmission by *Ix. ricinus* ticks and causing encephalitis in ovine species, but these viruses are restricted geographically. The most recent example of LIV-like infection was the detection of a virus causing encephalitis in goats in northern Spain (103). This was initially attributed to LIV due to genetic similarities to existing strains in the UK, but the virus has subsequently been renamed Spanish goat encephalitis virus (SGEV) based on differences across the complete genome (104) and its exclusive presence in Spain.

### Babesiosis

Babesiosis is a tick-borne intraerythrocytic protozoan disease that affects mammals and is caused by species within the genus *Babesia*. The disease presents with a range of signs. Many cases may be subclinical or show mild signs of low grade fever and anorexia that may be missed. However, clinical disease results from a combination of the host immune response and hemolytic anemia caused by destruction of erythrocytes. This can lead to hemoglobinuria (classically a port wine coloration in urine). In cattle the common name for the disease in the UK is redwater fever. Overt signs include a rapid onset fever reaching 41°C and non-specific signs including anorexia, depression and weakness. Death can result from hepatic and respiratory complications, and renal congestion caused by deposition of hemoglobin in the renal tubules. Following recovery, low levels of infection may be maintained within erythrocytes of affected animals for a number of years without signs of clinical disease and which may form a reservoir of infection for feeding ticks. Calves below 9 months of age demonstrate an innate, inverse, age related resistance, unrelated to maternal immunity, and do not suffer clinical disease.

**Figure 4:**
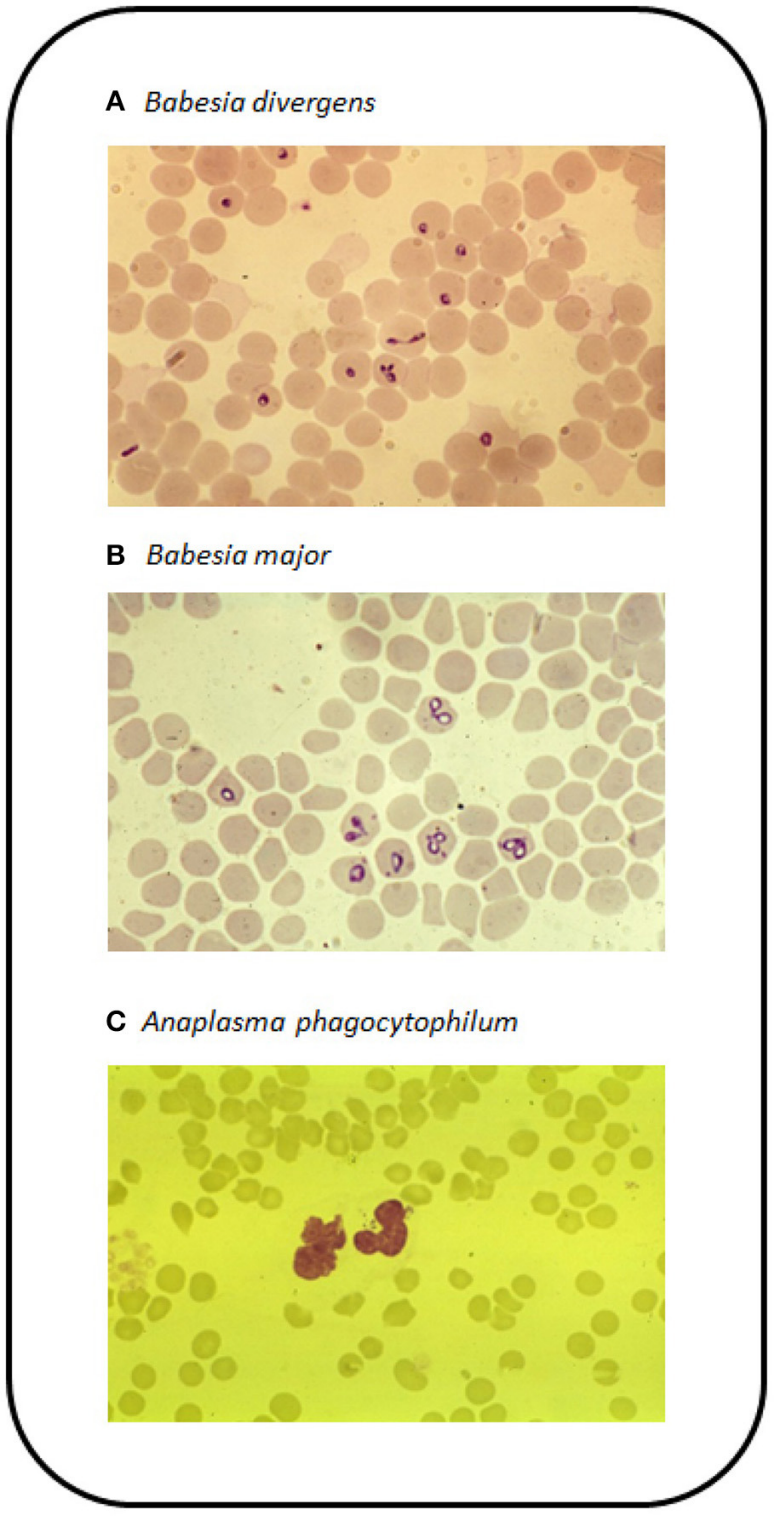
Blood films stained with Giemsa stain for **(A)** *B. divergens* in erythrocytes, **(B)** *B. major* in erythrocytes, and **(C)** *A. phagocytophilum* in the cytoplasm of neutrophils.

*Babesia* spp. only infect female ticks following blood feeding on infected animals and the parasites are transmitted via transovarial transmission to the next larval generation and subsequently to nymphal and adult ticks via transstadial transmission. Thus, at least one complete generation of ticks may be infected and are capable of transmitting the disease to naïve animals. Globally, the most significant species causing babesiosis in cattle are *B. bigemina* and *B. bovis* (105) with both being found on almost all continents. The most common species causing disease in Europe is *B. divergens* (Figure 4A), which is also the most widespread *Babesia* species affecting cattle in temperate regions and was first described in England by McFadyean and Stockman (106). It was originally named *Piroplasma divergens*, referencing the pear shaped paired merozoites lying at a typically divergent angle within the erythrocyte. Genetic evidence for the presence of *B. divergens* in British livestock has only recently been confirmed (107). Infections occur sporadically throughout Europe and may extend as far south as North Africa (108). Its distribution is defined by that of its tick vector, *Ix. ricinus*, which requires a microhabitat with at least 80% humidity to support metamorphosis and survival of life cycle stages off the host. This may include unimproved permanent pasture, rough moorland grazing, headlands and hedges of well-maintained pasture as well as forest floor. In addition *B. divergens* is zoonotic and has resulted in death in a number of humans, particularly in splenectomised or immunocompromised individuals (109). A second *Babesia* species has been detected in English cattle (110) transmitted by *H. punctata* in the South-east and based on its morphology is now considered to be the relatively non-pathogenic species *B. major* (111, 112) (Figure 4B). Additional species that can infect cattle include *B. bovis, B. bigemina, B. ovata* in Eastern Asia, *B. occultans* in Africa and more recently the Mediterranean area and *B. venatorum* (formerly *Babesia* sp. EU1) (105). Treatment may include supportive therapy including intravenous administration of fluids, blood transfusion and administration of vitamins as well as anti-protozoal chemotherapy using Imidocarb diproprionate.

A threat to equines in the UK is equine piroplasmosis caused by *Babesia caballi* or *Theileria equi* infection. Historically, the UK has been considered free of equine piroplasmosis despite the presence of seropositive and pathogen positive horses resident within the country (113) and populations of one of its tick vectors, *Dermacentor reticulatus*, being present in Wales and southern England (90). Nevertheless, the risk of causative pathogens becoming established within the vector population is evident and could lead to autochthonous transmission in the future.

A range of *Babesia* species cause mild disease in sheep and goats. These include *B. ovis, B. motasi*, and *B. crassa* (114). Of these, *B. motasi* has been detected in Wales (115, 116) and England (117), both are associated with *H. punctata* ticks. In Europe, disease presents as hemolytic anemia and chronic wasting, although it is rare.

Canine babesiosis is caused by a small number of piroplasms (see Table 3). Disease can be unapparent but in severe cases, dogs can develop fatal anemia (118). All canine-associated *Babesia* species are considered exotic to the UK. However, there have been a number of reports of individual dogs infected with *B. canis* and *B. vogeli* following travel in Europe (119–121). In 2015/2016 there were reports of autochthonous transmission of *B. canis* by *D. reticulatus* ticks in Harlow, southern England (122).

**Table 3:** Tick-borne diseases of livestock in Europe.

**Disease**	**Pathogen**	**Vector species**	**Susceptible species**	**Comment**
African Swine fever	African swine fever virus	*Ornithodoros* spp.	Pig	The vector is absent from Europe, transmission is directly from pig to pig.
Louping ill	Louping ill virus	*Ixodes ricinus*	Sheep Red grouse	Restricted distribution, mainly found within the British isles.
Babesiosis	*Babesia bigemina*	*Rhipicephalus bursa*	Cattle	
	*Babesia bovis*	*Rh. annulatus*	Cattle	
	*Babesia major*	*Haemaphysalis punctata*	Cattle	
	*Babesia divergens*	*Ix. ricinus*	Cattle	
	*Babesia occultans*	*Hy. Marginatum*	Cattle	
	*Babesia canis*	*Rh. sanguineus*	Dog	
	*Babesia vogeli*	*Rh. sanguineus*	Dog	
	*Babesia gibsoni*	*Rh. sanguineus*	Dog	
	*Babesia ovis*	*Rh. bursa*	Sheep	
	*Babesia motasi*	*Ha. punctata*	Sheep	
	*Babesia caballi*	Various tick species	Horse	
Theileriosis	*Theileria annulata*	*Hyalomma marginatum*	Cattle	
	*Theileria lestoquardi*	*Hyalomma* spp.	Sheep	
	*Theileria equi*	*Hyalomma* spp.	Horse	
	*Theileria orientalis*	*Ha. punctata*	Cattle	
		*Ha. punctata*	Sheep	
	*Theleria luwenshuni*	*Ha. punctata*	Sheep	
Anaplasmosis	*Anaplasma phagocytophilum*	*Ix. ricinus*	Cattle	
	*Anaplasma marginale*	*Ixodes* and *Rhipicephalus* spp.	Cattle	
Hepatozoonosis	*Heptatozoon canis*	*Rhipicephalus sanguineus* s.l.	Dog	Infection through ingestion of infected ticks

### Theileriosis

Theileriosis is a tick borne hemoparasitic disease of livestock including cattle, sheep, goats and equids caused by *Theileria* spp., which are apicomplexan protozoa closely related to *Babesia*. Unlike *Babesia sp*., transmission of *Theileria* spp. within the tick vector is transstadial only. Infection is acquired by larval or nymphal ticks feeding on infected animals and is maintained in the following nymphal and adult stages. No transovarial transmission of *Theileria* spp. has been demonstrated within tick vectors. Whilst both *Babesia* and *Theileria* spp. are transmitted through the bite of infected ticks, *Babesia* sp directly enter erythrocytes of infected animals whereas *Theileria* spp. initially undergo a lymphocytic phase of division (Schizogony) to produce merozoites which are released to invade host erythrocytes where further division occurs. Three species of *Theileria* cause significant economic impact on cattle farming worldwide, *T. parva* (East Coast Fever), *T. annulata* (Tropical Theileriosis), and *T. orientalis* (Far East, Australasia). Clinical bovine theileriosis is mainly reported from The Middle East, Africa, Asia (123) and most recently in Australia and New Zealand (124). East Coast Fever caused by *T. parva* is the most severe form of disease in cattle presenting with fever and enlarged lymph nodes, particularly near tick bites (125). Other disease signs include anorexia, nasal discharge, and diarrhea with mortality reaching 100% during severe outbreaks. Blood smears show the presence of parasite in both leukocytes and in erythrocytes.

A benign form of *Theileria* has been detected in cattle in southern England transmitted by the tick *H. punctata* (126). Based on morphology of the parasite in blood smears it was identified as *T. mutans*. However, serology suggested that it was identical with *Theileria sp*. from Asia (127). Recently, there has been further evidence of *Theileria* species present in UK through the detection of the parasite in the blood meal of mosquitoes that have fed on cattle (128) grazing a known site of *H. punctata* activity. This was identified as *T. orientalis* based on genomic sequence data and although *T. orientalis* strains cause severe disease in cattle in Asia and Australasia, there have been no records of clinical bovine theileriosis in the UK. Ovine theileriosis caused by *Theileria luwenshuni* has been reported in North Kent associated with high tick burden (117).

### Anaplasmosis (Tick Borne Fever, Pasture Fever)

Tick-borne fever was recognized as a discrete disease of cattle in the late 1940's (129). The causative agent is a gram negative bacterium now known as *Anaplasma phagocytophilum* (130) a name that has replaced three synonyms, *Cytoecetes phagocytophila, Erhlichia phagocytophila, Ehrlichia equi* and is the causative agent of human granulocytic anaplasmosis (HGA). As the common disease name suggests, infection presents as a fever and anorexia. There have been repeated reports of tick-borne fever in dairy herds in the UK (131, 132) and reduction in milk yield can indicate infection. In more serious cases, abortion and stillbirth are signs of disease (133). Some animals are also affected by respiratory distress in response to infection.

In Europe *A. phagocytophilum* is transmitted by the sheep tick *Ix. ricinus*, so like *B. divergens*, its occurrence is dictated by the presence and abundance of this tick species. The disease has been reported from across the UK and Ireland. In continental Europe, cases have been reported from Spain, France, Germany and the Scandinavian countries. *Anaplasma phagocytophilum* is also present in North America and transmitted by ticks, such as *Ix. scapularis* and is more commonly identified as a cause of HGA (134). Cases of HGA in Europe are rare but do occur, often as a mild fever (135). Most outbreaks occur following the introduction of naïve cattle onto tick-infested fields (136). Following feeding by an infected tick, the bacteria are detectable in circulating granulocytes, particularly neutrophils (Figure 4C). This coincides with the onset of fever (>40°C). Due to the infection of granulocytic cells, infected animals become immunosuppressed and this can lead to increased susceptibility to other infections, such as tick pyaemia caused by *Staphylococcus aureus* (137). This can be particularly devastating in sheep herds (138). Treatment is typically based around the administration of oxytetracycline or sulfamethazine.

## Emerging Tick-Borne Threats in Europe and Africa

A significant emerging threat to the pig production industry has been the emergence of African swine fever virus (ASFV) in Europe. The virus evolved in Africa where it is transmitted by soft ticks within the genus *Ornithodoros*. Infection in native species, such as warthogs (*Phacocherus africanus*) causes subclinical disease, whereas infection in domestic pigs can be devastating with mortality reaching 100% in some cases (139). ASFV was introduced in the Caucasus region in 2007 and spread rapidly north into the Russian Federation, presumably through movement of livestock. It then entered the wild boar population in the Baltic States and from there emerged in Western Europe in the summer of 2018 (140, 141). In Northern Europe there are no known tick vectors, so transmission is through direct contact between animals which has resulted in the culling of wild boar populations in an attempt to reduce disease spread.

Additional exotic threats include infection with Crimean-Congo hemorrhagic fever virus (CCHFV) and Nairobi sheep disease virus (NSDV). The former is transmitted by *Hyalomma* ticks to livestock that can become infected, but do not show signs of disease. The main risk is to humans that have contact with infected meat or milk, as this may lead to fatal haemorrhagic fever (142). CCHFV has a wide distribution from Spain and the Balkans in Europe, Africa and Asia (143). Nairobi sheep disease is a potentially fatal disease of ovines and found in parts of Africa where *Rhipicephalus* ticks are active (144). A variant of NSDV, Ganjam virus has been reported in India, although this represents no immediate threat to the UK.

## Risks from Exotic Ticks

The introduction and establishment of exotic ticks could lead to a change in the current risk assessment of animal diseases due to tick-borne pathogens. This could lead to either the direct introduction of a pathogen with the ticks or provide a reservoir population should a pathogen be brought in by an infected vertebrate. A range of pathways for introduction exist, perhaps the most important being those enabled by humans. The importation of ticks on animals, such as dogs, have been well-documented for *Rh. sanguineus* (3, 145) and can lead to infestation of houses. A further risk associated with this tick species is the potential for introduction of *Hepatazoon canis*, a common disease of dogs in southern Europe resulting from ingestion of infected ticks. There have been a number of recorded cases in the UK (146). However, expert opinion and surveillance suggest that this tick species cannot persist in the British climate at present. Another pathway is the introduction of ticks on migrating birds. A number of studies have reported *Hyalomma spp*. on birds migrating north through Europe (147, 148). Further studies have confirmed that such ticks can be infected with zoonotic pathogens (149), such as Crimean-Congo haemorrhagic fever virus. But again, it is unlikely that such ticks will survive and thrive in the UK, so onward transmission of pathogens will be limited. A recent report has suggested that the presence of an adult *H. rufipes* found on an untraveled horse in the south of England could have been introduced as a nymph by migrating birds (150). This is of concern as it suggests partial completion of the ticks' lifecycle within the UK.

## Midges and Midge-Borne Diseases

**Figure 5:**
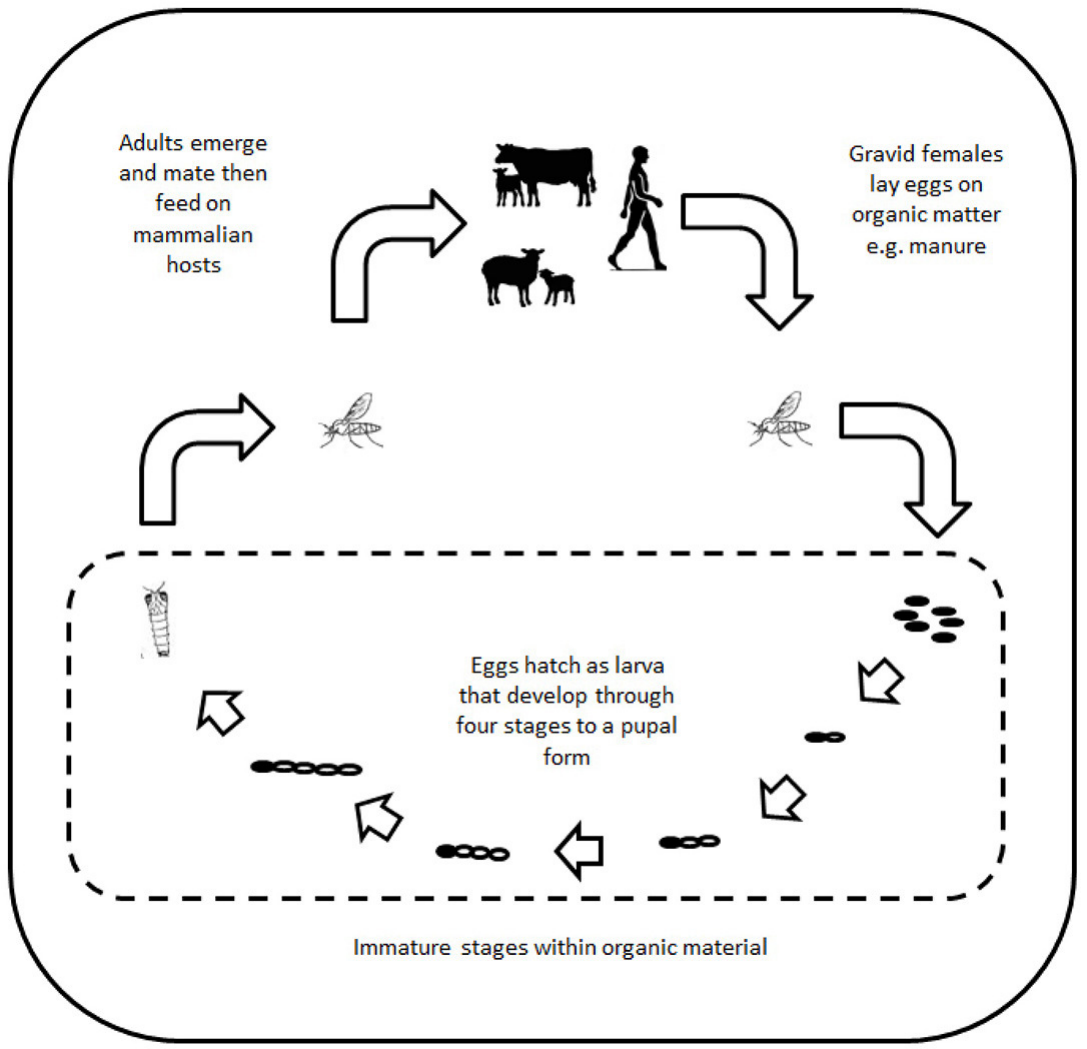
Schematic showing the life cycle of *Culicoides* midges.

Biting midges (Figure 5) within the genus *Culicoides* (Latreille, 1809) are the vectors of a number of significant diseases of livestock including bluetongue virus and African horse sickness virus (AHSV). Species within the genus are small, ranging from 1 to 3 mm in length and so morphological identification can be challenging, and with over 1,000 species within the genus a comprehensive classification is not currently available (151). However, within Europe the main species identified as responsible for virus transmission are *C. obsoletus, C. scoticus, C. dewulfi, C. chiopterus, C. pulicaris*, and *C. punctatus* (152). Whilst mosquitoes and ticks can be introduced by human interventions including cars, freight lorries, shipping, and migratory animals, midges can be moved over large distances by wind movements (153). This mechanism has been responsible for the introduction of a number of exotic livestock viruses in the UK (154). The following midge-vectored viruses represent those that have either caused disease outbreaks in recent years, or have the potential to do so if introduced, to the UK. Bovine ephemeral fever is included in this section, although there is still uncertainty over the role of midges and mosquitoes in transmitting this virus (155).

### Bluetongue

Bluetongue is a midge-borne disease caused by serotypes of the *Orbivirus*, bluetongue virus (BTV). Ruminants are susceptible to disease with cattle presenting with elevated temperature and congestion of, and discharge from, the mucous membranes. This can develop into crusts and erosion of the nasal and oral mucosa. Animals can become lame due to coronitis, inflammation of the coronary band above the hoof, and ulceration of the teats can occur. Transplacental transmission can lead to congenital deformities and developmental defects in live births (156). Diagnosis is based on serology and detection of virus using RT-PCR (157). Critically BTV serotype 8 emerged in the Netherlands in 2006 (158) and proceeded to spread across Europe. Cases of BTV infection were reported in England in 2007, likely the result of airborne spread of the midge vector across the North Sea. As a notifiable disease, control measures were introduced that eliminated the disease in the UK. In 2015, BTV serotype 8 re-emerged in France and has persisted over two winters (159). Despite its proximity, the potential of transmission to the UK is considered to be low.

### Schmallenberg

Schmallenberg virus (SBV) was first reported in a herd of cattle in Germany experiencing a drop in milk yield and diarrhea (160). Infection with SBV in adult ruminants can be mild but infection *in utero* can lead to malformation and abortion, and this is usually how the disease presents. The virus is an *Orthobunyavirus* and is transmitted by *Culicoides* biting midges. SBV spread rapidly across Europe and the first case of disease reported in England occurred in East Anglia in April of 2012. Subsequently, there were repeated outbreaks of SBV infection in both cattle and sheep in England (161). Although currently there are no active UK outbreaks, the threat of disease remains high. Malformation in new born animals is typical of SBV infection including contraction of the limbs, arthrogryposis, and microencephaly. The diagnosis can be confirmed by detection of virus by RT-PCR (162) or detection of SBV antibodies in the mother (163).

### African Horse Sickness

African horse sickness is characterized by a sudden onset fever and edema of the head and neck. A more severe form is associated with pulmonary illness that leads rapidly to death. Mortality rates can reach as a high as 70%. The main vector for transmission is *C. imicola*, a species absent from northern Europe. The disease is caused by another *Orbivirus*, African horse sickness virus (AHSV) that is endemic in tropical and sub-tropical regions of Africa. Outbreaks in Europe have occurred, most notably the introduction of AHSV in a consignment of zebras brought into a safari park near Madrid in 1987 causing the disease to persist in the Iberian Peninsula until 1990 (164).

### Bovine Ephemeral Fever

Bovine ephemeral fever or three-day sickness is caused by infection with bovine fever ephemerovirus (BEFV—formerly bovine ephemeral fever virus). Infection causes transient fever with ocular and nasal discharge, depression and recumbency (165). Severe disease can lead to livestock deaths and recent outbreaks in Israel and Turkey have reported significant mortality (166, 167). The virus is transmitted by arthropod vectors although an exact association with a particular species has not been established, with BEFV being detected in both *Culicoides* midges and mosquitoes in Australia (165). Bovine ephemeral fever is either enzootic or occasionally epizootic in Africa, Asia and Australia (168–170). There have been no cases reported from Europe, with the exception of possible cases in the European region of Turkey, so currently there is a low risk of its emergence in the UK.

## Discussion

**Figure 6:**
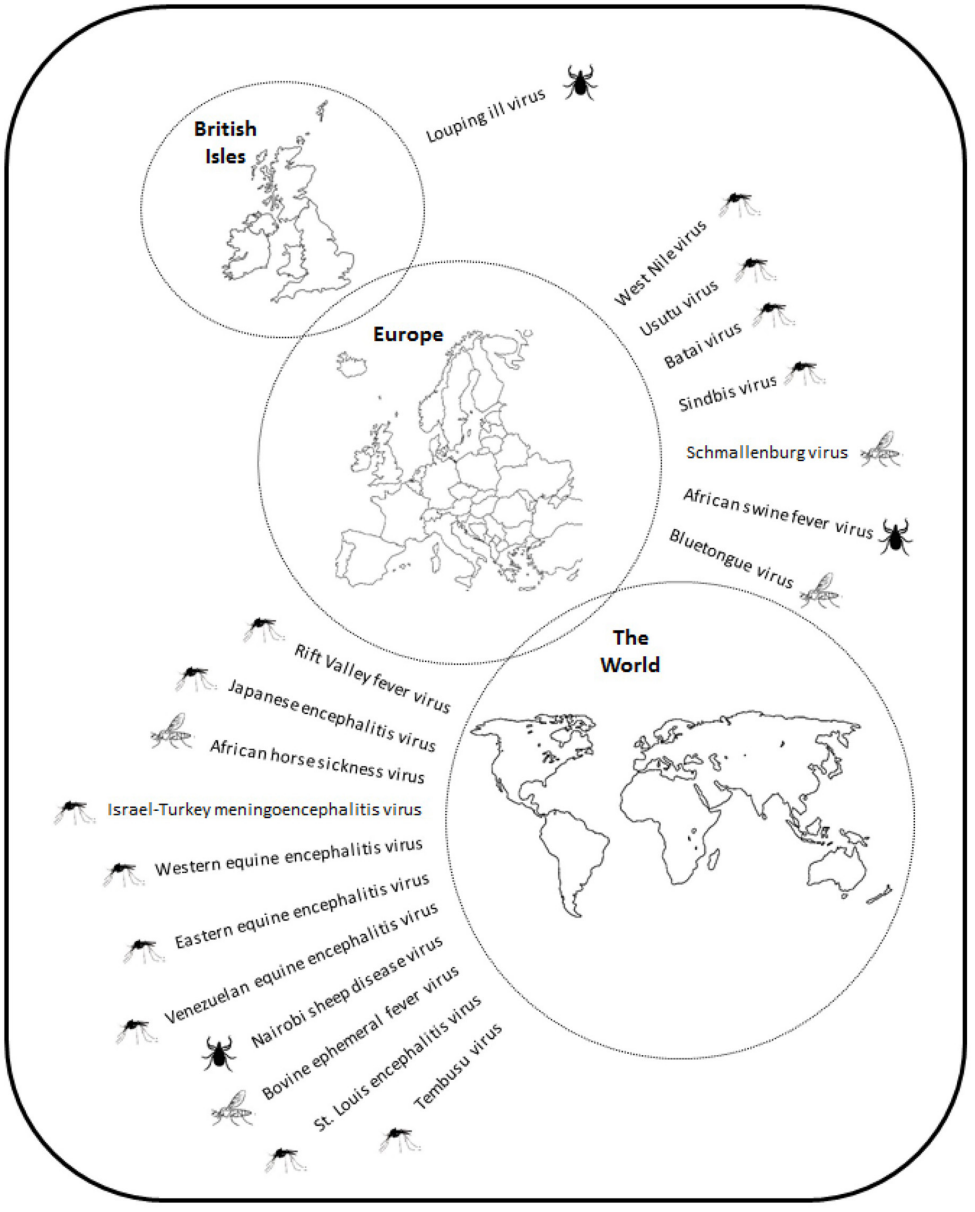
Schematic showing the distribution of virus pathogens of animals in the British Isles, Europe and the World.

This review has highlighted a large number of pathogens that infect animals in the UK, and others that are at risk of introduction (Figure 6). A striking feature of this extended list is the small number of arthropod-borne viruses that are currently present in the UK, limited to one tick-borne virus, LIV. Various reasons for this have been suggested including a low level of competence of indigenous species (171) and climatic factors, such as lower mean temperatures and a shorter active season for vectors than in other parts of Europe. However, a growing number of studies have shown that indigenous species of mosquitoes are capable of transmitting viruses under experimental conditions similar to that found during the summer months (10, 12, 45). Another key factor is the absence of certain vector species, for example sandflies and tick species*. Phlebotomus* sandflies transmit *Leishmania infantum* to dogs causing canine leishmaniosis in southern Europe. The absence of sandflies in the UK means that there is no vector borne transmission, although infected dogs are imported (172) and there is suspicion that dog to dog transmission can occur (173). The introduction of exotic ticks, such as *Hyalomma* and *Rhipicephalus spp*., vectors of viruses, such as CCHFV and NSDV, respectively, could lead to the introduction and establishment of these diseases. In the case of *Hyalomma* species, ticks are being introduced by migrating birds but have so far failed to establish a detectable reproductive population. Changes to the climate may make the UK more permissive for such species. Increases in a range of parameters will influence vector populations. For example, an increase in mean temperatures throughout the year will extend the period over which vectors are active and lead to extended periods where temperatures are permissive for virus replication, which may promote vector competence. Another area of uncertainty is the ability of pathogens to survive the winter. An increase in midwinter temperatures will promote vector survival and increase the probability that infected vectors will enable overwintering of pathogens. Critically, WNV is successfully overwintering in Central Europe, which is driving repeated outbreaks of disease and increasing its distribution. For anthropophilic mosquitoes, such as *Ae. albopictus*, climate modeling suggests that only the southeast of England has suitable climatic conditions for the mosquito species to establish, coincidentally the one part of the UK where *Ae. albopictus* has been detected. The current trends in climate change, particularly to the daily temperature range will increase the areas of the UK that can accommodate the species (174).

In addition to climate change, human action in activities, such as international travel by air, livestock movements and conversion of land to agricultural use can lead to the movement of disease vectors (175, 176). The introduction of *Ae. albopictus* into southern England may have been introduced by cars or lorries entering the country. Whilst the introduction of an anthropophilic mosquito species may not change the risk of disease transmission to animals, the addition of an invasive tick may be more significant, particularly as there are already tick-borne diseases active in the UK. The emergence of the Asian long-horned tick, *Haemaphysalis longicornis*, in North America is a dramatic demonstration of how quickly an invasive tick species can establish in a new environment. As its common name suggests, the tick is a native of East Asia and had been repeatedly intercepted on quarantined livestock entering the United States. However, in 2017 it was detected in a sheep flock in New Jersey (177). Subsequent surveillance confirmed the presence of the tick in a further eight US states. Modeling has suggested that the tick could eventually spread across much of the US and Mexico (178). Of significant concern is the ability of this species to transmit a number of diseases including severe fever with thrombocytopenia syndrome in humans (179) and Theileriosis in livestock (180), in addition to transmitting existing tick-borne diseases already present in the US.

The implication of these observations are that surveillance for the introduction and spread of invasive arthropod species is necessary to offer an opportunity to prevent establishment and to predict at-risk areas well before a pathogen is introduced. Allied to this is an understanding of the assemblage, behavior, ecology and abundance of indigenous vectors. The monitoring of the mosquito species *Culex modestus* in the UK, is an example of this (181). Genetic studies suggest that this species is a recent introduction from continental Europe (182). The population has expanded across large areas of the Thames Estuary and East Anglia, and these areas are now considered at greater risk of WNV spread, were the virus to be introduced. Monitoring for other invasive arthropod vectors, such as ticks and sandflies will provide an early warning for increases in the risk of arbovirus emergence. This reflects trends observed in southern Europe where there is push for harmonization between governments in response to an increasing risk to public health (183). In addition to field-based surveillance for vector species, there are initiatives in the UK to introduce innovative measures to detect changes to vector distribution, prevalence on animals and incidence of disease (184, 185).

## Conclusions

A key driver in the application of surveillance of livestock, domestic pets, and wildlife is to detect disease before there is widespread transmission of disease. This reduces the risk of spill-over of some diseases into the human population, ameliorates the economic impact of the outbreak to industry, reduces potential harm to domestic animals and limits challenges to biodiversity within wildlife. The cost of surveillance needs to be proportionate to the risk and balanced against the cost of an outbreak. The estimated cost to the agricultural sector as a result of the 2001 outbreak of foot and mouth disease in the UK was £3.1 billion with the tourism sector being equally affected (186). It is unlikely that the introduction of an exotic arbovirus disease will be as costly in strict financial terms as this, although the ability to eliminate the disease will be highly dependent on a range of factors, including competent vector distribution and the movement of compromised animals. The experience from mainland Europe is that once established, vector-borne diseases with a wildlife reservoir are difficult to eliminate and subsequent emergence is unpredictable and challenging to control. The early identification of the source of the introduction, controlling infected vector populations and the availability of effective interventions, such as vaccination, all help to reduce the impact of disease but not continued transmission. For many disease-vector combinations, these interventions will be difficult to implement and thus elimination may not be possible and control will be replaced by prevention. The UK is in a fortunate position with respect to vector-borne diseases in animals due in part to geographical barriers. However, with changes in vector and pathogen distribution this is likely to change in the coming decades.

## Author Contributions

NJ conceived the idea. AF, DD-R, LH-T, LP, and NJ wrote the manuscript.

### Conflict of Interest

The authors declare that the research was conducted in the absence of any commercial or financial relationships that could be construed as a potential conflict of interest.
