# Supplementary material for: Emerging Threats to Animals in the United Kingdom by Arthropod-Borne Diseases
Source: Front Vet Sci. 2020 Feb 4;7:20. doi: 10.3389/fvets.2020.00020 (PMC7010938; doi:10.3389/fvets.2020.00020)
Supplement: Supplementary file 1 [file Table_1.DOC]

**Table S1: Bionomics of UK Mosquito Species**

| **Species^1^** | **Occurrence in UK^1^** | **Active (Adults)** | **Annual Generations (UK)** | **Feeding Preference** |
| --- | --- | --- | --- | --- |
| *Aedes cinereus* | Widespread, patchy | June – Aug^2^ | Univoltine^1^ | M^3^ |
| *Aedes vexans* | Sporadic reports | No Data | No Data | M^3^ |
| *Anopheles algeriensis* | Few reports | No Data | No Data | No Data |
| *Anopheles claviger* | Widespread | Mar – Oct^3^ | Multivoltine^3^ | M^3^ |
| *Anopheles messeae* | Widespread | May – Oct^3^ | Multivoltine^3^ | M^4^ |
| *Anopheles atroparvus* | Widespread | May – Oct^3^ | Multivoltine^3^ | M^4^ |
| *Anopheles daciae* | Few reports | No Data | No Data | No Data |
| *Anopheles plumbeus* | Widespread | Apr – Oct^2^ | Bivoltine^2^ | M |
| *Coquillettidia richiardii* | Widespread | Apr – Aug^2^ | Univoltine^3^ | M^3^ |
| *Culex modestus* | South East England | Jul – Sept^2^ | No Data | M^3^ / O^4^ |
| *Culex pipiens s.s.* | Widespread, abundant | Apr – Nov^2^ | Multivoltine^3^ | O^3^ |
| *Culex pipiens, biotype molestus* | Few reports | All year round^2^ | Multivoltine^3^ | M^3^ |
| *Culex torrentium* | Widespread | Apr – Nov^2^ | Multivoltine^3^ | O^3^ |
| *Culex europeaus* | Few reports | No Data | No Data | No Data |
| *Orthopodomyia pulcripalpis* | Few reports (SE England) | July – Sept^3^ | Univoltine^3^ | O^3^ |
| *Culiseta longiareolata* | Few reports | No Data | No Data | No Data |
| *Culiseta alaskaensis* | Few reports | No Data | No Data | No Data |
| *Culiseta annulata* | Widespread | All year round^2^ | Multivoltine^3^ | M^5^ / O^3^ |
| *Culiseta fumipennis* | Widespread (SE England) | No Data | No Data | No Data |
| *Culiseta litorea* | Widespread (SE England) | Apr – Aug^2^ | No Data | No Data |
| *Culiseta morsitans* | Widespread | May – Sept^2^ | Univoltine^3^ | O^3^ |
| *Culiseta subochrea* | Rare | No Data | No Data | No Data |
| *Finlaya geniculatus* | Widespread (England) | No Data | No Data | No Data |
| *Ochlerotatus annulipes* | Widespread | Apr – Sept^3^ | Univoltine^3^ | M^3^ |
| *Ochlerotatus cantans* | Widespread | Apr – Sept^2^ | Univoltine^3^ | M^3^ |
| *Ochlerotatus caspius^6^* | Rare | Apr – Oct^2^ | Multivoltine^3^ | M^3^ |
| *Ochlerotatus communis* | Few reports | Do Data | No Data | No Data |
| *Ochlerotatas detritus* | Widespread | Mar – Nov^2^ | Multivoltine^3^ | M^3^ |
| *Ochlerotatus dorsalis* | Rare | May – Sept^3^ | Multivoltine^3^ | M^3^ |
| *Ochlerotatus flavescens* | Rare | May – Aug^3^ | Univoltine^3^ | M^3^ |
| *Ochlerotatus leucomelas* | One report | No Data | No Data | No Data |
| *Ochlerotatus punctor* | Widespread | Mar – Oct^2^ | Univoltine^3^ | M^3^ |
| *Ochlerotatus sticticus* | Few reports | No Data | No Data | No Data |
| *Ochlerotatus rusticus* | Widespread | Apr – Sept^2^ | Univoltine^3^ | M^3^ |

**References**

^1^Medlock, J.M., Snow, K.R. & Leach, S. 2007. Possible ecology and epidemiology of medically important mosquito-borne arboviruses in Great Britain. Epidemiology and Infection 135, 466-482.

^2^Brugman, V.A., Horton, D.L., Phipps, L.P., Johnson, N., Cook, A.J.C., Fooks, A.R. & Breed, A.C. 2013. Epidemiological perspectives on West Nile virus surveillance in wild birds in Great Britain. Epidemiology and Infection 141, 1134-1142.

^3^Snow, K.R. 1990. Mosquitoes. Richmond Publishing Company Ltd. Slough, England.

^4^Brugman, V.A., Hernandez-Triana, L.M., Prosser, A.W.J., Weland, C., Westcott, D.G., Fooks, A.R. & Johnson, N. 2015. Molecular species identification, host preference and detection of myxoma virus in the Anopheles maculipennis complex (Diptera: Culicidae) in southern England, UK. Parasites & Vectors 8, 421.

^5^Fernández de Marco, M., Brugman, V.A., Hernández-Triana, L.M., Thorne, L., Phipps, L.P., Nikolova, N.I., Fooks, A.R. & Johnson, N. 2016. Detection of *Theileria orientalis* in mosquito blood meals in the United Kingdom. Veterinary Parasitology 229, 31-36.
