# Supplementary material for: Emerging Threats to Animals in the United Kingdom by Arthropod-Borne Diseases
Source: Front Vet Sci. 2020 Feb 4;7:20. doi: 10.3389/fvets.2020.00020 (PMC7010938; doi:10.3389/fvets.2020.00020)
Supplement: Supplementary file 2 [file Table_2.DOC]

**Table S2: List of UK tick species (from Arthur, D.R. 1963. British ticks. Butterworths, London, UK)**

| **Genus** | **Species** | **Principle host** |
| --- | --- | --- |
| *Ixodes* | *ricinus* | Mammals and birds |
|  | *festai* | Rabbits (*Oryctolagus cuniculus*) |
|  | *caledonicus* | Birds |
|  | *hexagonus* | Hedgehogs (*Erinaceus europaeus*) |
|  | *canisuga* | Foxes (*Vulpes vulpes*), badgers (*Meles meles*), domestic dogs |
|  | *lividus* | Sand martins (*Riparia riparia*) |
|  | *arboricola* | Small mammals and birds |
|  | *dorriensmithi* | Shrew (*Crocidura cassiteridum*) |
|  | *arvicolae* | Water vole (*Arvicola amphibius*) |
|  | *guernsayensis* | Bank vole (*Myodes glareolus*) |
|  | *pari* | Birds |
|  | *unicavatus* | Birds |
|  | *rothschildi* | Birds |
|  | *vespertilionis* | Bats |
|  | *trianguliceps* | Shrews and rodents |
|  | *uriae* | Marine bird |
| *Dermacentor* | *reticulatus* | Mammals |
| *Haemaphysalis* | *Punctata* | Mammals |
| *Argas* | *reflexus* | Birds |
|  | *vespertilionis* | Bats |
| *Ornithodoros* | *capensis* | Marine birds |
